# Felt Stigma and Its Underlying Contributors in Epilepsy Patients

**DOI:** 10.3389/fpubh.2022.879895

**Published:** 2022-04-26

**Authors:** Lingyan Mao, Keying Wang, Qianqian Zhang, Jing Wang, Yanan Zhao, Weifeng Peng, Jing Ding

**Affiliations:** ^1^Department of Neurology, Zhongshan Hospital, Fudan University, Shanghai, China; ^2^Teachers College, Columbia University, New York, NY, United States

**Keywords:** epilepsy, stigma, QOL, diffuse tensor imaging, neuroticism

## Abstract

**Objective:**

To explore the correlated clinical and psychological factors of stigmatization and investigate the relationship between stigma and white matter abnormalities in epilepsy patients.

**Methods:**

Stigmatization was obtained by a three-item stigma scale in 256 epilepsy patients with genetic or unknown etiology. Personality and quality of life (QOL) were assessed by Eysenck Personality Questionnaire (EPQ) and QOL-31 questionnaire respectively. One hundred and fourteen of them were performed Hamilton Depression Scale-17 (HAMD) and scanned with diffusion tensor imaging in 3T MRI. Fractional anisotropy (FA) values of frontotemporal contact fibers were calculated.

**Results:**

There were about 39.8% patients felt stigma, with the highest score (Score 3) in 8.2% (21/256). Stigma scores were significantly negatively correlated with education (*P* < 0.01), age of onset (*P* < 0.05), extraversion score of EPQ (*P* < 0.01), total and all the subscale QOL scores (*P* < 0.001), and positively correlated with duration (*P* < 0.01), HAMD score (*P* < 0.001), neuroticism score of EPQ (*P* < 0.001). We found negative correlation between stigma scores and FA values of right superior longitudinal fasciculus and left cingulum (*P* < 0.05). Logistic regression results showed that FA value of left cingulum (*P* = 0.011; OR = 0.000), social function (*P* = 0.000; OR = 0.935) of QOL, and neuroticism score of EPQ (*P* = 0.033; OR = 1.123) independently correlated to felt stigma.

**Conclusion:**

Felt stigma in epilepsy patients was found to be correlated with neuroticism, depression, and deficient social function of QOL, which might be predisposed by the impairment of the left cingulum. Our results provide preliminary evidence for the underlying neural circuits in stigmatization.

## Introduction

Epilepsy is a common neurological disease characterized by epileptic seizures. About 1% of people worldwide (50 million) have epilepsy, and nearly 80% of cases occur in developing countries (1). Epilepsy can have adverse effects on social and psychological wellbeing. These effects may include social isolation, stigmatization, or disability. Stigmatization is a major contributor to the burden associated with the condition. It can also affect the families of those with the disorder (1, 2).

In many parts of the world, people with epilepsy and their families suffer from stigma. Goffman first explored the notion of epilepsy as a stigmatized illness in the early 1960's (3). It was defined as “a distinguishing mark (especially) of disgrace” or as “an attribute that is deeply discrediting.” Jacoby further explored the concept and distinguished “enacted stigma” from “felt stigma” (4). “Enacted stigma” refers to episodes of discrimination against people with epilepsy, solely on the grounds of their social unacceptability; whereas “felt stigma” refers to the shame associated with being epileptic and the fear of enacted stigma (4). In the studies of prevalent epilepsy, up to 50% of individuals reported experiencing epilepsy-related stigma (5, 6). In China, about 9 million people with epilepsy, and that 63% are untreated or not properly treated (7). More than 40% of people with epilepsy did not tell others about their disease, and 36% thought that people treated them differently because of their epilepsy (8). Concealment of epilepsy, concerns related to social life, and concerns related to future occupation were found as the predictors of felt stigma (6), which refers to the expected discrimination and shameful feelings that stop people from seeking help or sharing their experiences with others (9).

Stigma can affect people economically, socially, and culturally, including the education for children and adolescents, and work opportunities for adults (1, 10, 11). Felt stigma can reduce the quality of life even when seizures are well controlled (12, 13). In a study of 250 patients with epilepsy, 20% were unemployed (14). In Asia, epilepsy is a common negative reason for marriages and pregnancy (1, 10). Reducing stigma—helping patients “out of the shadows” is currently a major focus of activity of epilepsy support groups globally (15, 16).

Studies reported the public or patients' attitudes toward epilepsy (17). Only certain aspects of knowledge have an impact on the underlying cause of stigma in epilepsy. Previous studies demonstrated that feelings of stigma were associated with seizure severity (18), patient's age and seizure classification (mesial temporal lobe epilepsy) (19, 20). Felt stigma was also found to correlate with psychosocial aspects of patients' lives, including culture (15), education gap (21), lack knowledge of their diseases (22), lower household wealth (18), married status (23), depressive and social anxiety (24–27), etc. The social discrimination and felt stigma of epilepsy patients may be improved to a certain extent with the improvement of society's attitude toward epilepsy, despite far more struggle to fill the gap of culture, income, etc. It remains considerable efforts on the understanding of the underlying neurobiology of inner perception in epilepsy patients, which may provide new insights on the treatment of stigmatization in epilepsy.

With the development of neuroimaging technology, the underlying organic impairment of psychiatric symptoms in epilepsy can be detected. Kavanaugh et al. (28) suggested white matter integrity within frontotemporolimbic (FTL) and non-FTL regions correlates with depressive symptomatology in temporal lobe epilepsy. In our previous study, we found that the functional metabolism of the hippocampus was a risk factor relating to depressive symptoms in patients with epilepsy (29). Furthermore, abnormalities in the integrity of the white matter can be detected by diffusion tensor imaging (DTI). Mao et al. (30) found significantly lower mean FA values compared with healthy controls in the bilateral frontotemporal contact fibers, such as uncinate fasciculus, cingulum bundle, arcuate fasciculus, and the right arcuate fasciculus were independent risk factors of psychoticism in epilepsy patients. These all provided important insights into the pathophysiological mechanisms in epilepsy.

Previous researchers studied the incidence and correlated factors of stigma in all epilepsy patients, including seizures secondary to or comorbid other diseases. This study aims to display (1) the incidence of felt stigma in epilepsy patients with genetic and unknown etiology in China; (2) clarify clinical and psychosocial factors associated with felt stigma in epilepsy; (3) further identify the neural correlates of felt stigma in patients with epilepsy by DTI.

## Materials and Methods

### Subjects

A total of 256 epilepsy patients who were diagnosed according to 2017 classification and Terminology of the International League against Epilepsy (ILAE) (31) were enrolled from June 2009 to October 2014 at the Department of Neurology, Zhongshan Hospital, Fudan University, Shanghai, China. One hundred and fourteen of them performed Hamilton Depression Scale (HAMD) and DTI scan. A patient was included: (1) if the patient aged from 16 to 65 years, (2) if the patient appeared normal on conventional brain MRI, (3) if the patient did not have any disease other than epilepsy, and (4) if the patient did not have a family history of psychiatric illnesses.

The study protocol was approved by the local institutional review board at the author's affiliated institutions. Written informed consent was obtained from each study participant.

### Clinical Characteristic

A clinical questionnaire including age, gender, and length of education was applied. Mini-mental state examination (MMSE) (32) tests were administered at the initial visit.

Socioeconomic status information was obtained from each participant including education, employment, income, and insurance. The income level was divided into two groups according to the local minimum living guarantee standard.

The duration of epilepsy, the type and frequency of seizures, and drug treatment history were elicited and recorded. Seizure severity was evaluated by the National Hospital Seizure Severity Scale (NHS3) (33).

### Social Stigma, Quality of Life and Psychological Assessment

An 18-item Brief Psychiatric Rating Scale (BPRS) was performed for psychiatric evaluation. Twenty-one of them scored over 35 on BPRS and were referred to psychiatrists for consultation, and none of them were diagnosed with psychosis.

Felt Stigma was assessed by a three-item scale, developed originally for use in stroke patients (34), and subsequently adapted for use in epilepsy (35). Patients provided a simple yes/no response to indicate whether they feel other people are uncomfortable with them, whether other people treat them like an inferior person, and whether other people would prefer to avoid them because of epilepsy. Respondents whose score on the scale is zero are deemed not to feel stigmatized. The higher the positive score, the more stigmatized respondents are considered to feel. Previous studies showed that the stigma scale is a reliable and valid measure (36, 37). The stigma scale was translated to Chinese with words that people with various education levels could understand.

The patients were assessed by 17-item HAMD to recognize depressive symptoms. The range of the scale scores is from 0 to 54. The Chinese version of HAMD had been tested and proved to have good validity and reliability (38).

We adapted the QOL-31 questionnaire to evaluate the epilepsy patient's quality of life (39). This questionnaire contains 31 questions and seven subscales; each of the subscales assesses a different domain of QOL: seizure worry, overall quality of life, emotional wellbeing, energy/fatigue, cognitive function, medication effects, and social function. The Chinese version has previously been demonstrated good reliabilities (alpha = 0.94) and validity. The reliabilities for the individual subscales were observed with good test-retest (0.87–0.97) and internal consistency (0.58–0.88). Higher scores in the QOLIE-31 are indicative of a better QOL.

Personality disorder traits were assessed by the Chinese version of the Eysenck Personality Questionnaire (EPQ) (40), consisting of four categories of temperament: psychoticism (P), extraversion (E), neuroticism (N), and the lie (L) scale.

### MRI Acquisition and Data Processing

MRI was performed on a General Electric 3T EXCITE HD scanner with a standard eight-channel phased array head coil. A standard protocol of conventional imaging was performed to measure conventional sagittal and axial T2-weighted fast spin-echo and coronal T1-weighted spin-echo imaging sequences. In all epilepsy patients, MRI scans were obtained in the interictal period (at least 1 week after the last seizure).

### DTI Protocol and Data Analysis

Using a single-shot spin-echo echo planar image (SE-EPI) sequence, DTI data without diffusion weighting (b = 0) were acquired simultaneously in 25 non-collinear directions (b = 1,000 s/mm2). Moreover, 22 contiguous slices were acquired with a 3 mm slice thickness and with no gap. They were performed using acquisition with a 128 × 128 matrix; and a 256 x 256 mm field of view (FOV). The other acquisition parameters were: repetition time (TR) = 6,000 ms; echo-time (TE) = 76.2 ms; number of excitations (NEX) = 2.

DTI data were processed using the software FuncTool (GE Healthcare). The principal 3D orientation of the major eigenvector was color-coded per voxel. Each diffusion tensor was sampled 6 times to optimize the signal-to-noise ratio (SNR). Isotropic diffusion-weighted, ADC, and fractional anisotropy (FA) maps were generated. By positioning the region of interest (ROI) (33 ± 0.4 mm2) the targeted fibers, we got the FA values of the bilateral arcuate fasciculus (AF), uncinate fasciculus (UF), cingulum bundle (CB), fornix, superior longitudinal fasciculus (SLF) and anterior commissure (AC) as we previously described (30).

### Statistical Analysis

Numerical variables are expressed as mean ± standard deviation (s.d.) or median [interquartile range (IQR)]. Statistical analysis was performed using the SPSS software version15.0 (SPSSInc., Chicago, IL). Differences were made by χ*2* test for categorized data, an unpaired Student *t*-test for continuous data that were roughly normally distributed. Correlation between stigma scores and clinical characteristics, using Spearman correlation and logistic regression test. We used forward conditional methods to screen variables, which were included at a level of 0.05 and excluded at a level of 0.1. All tests were two-sided with a significance level of *P* < 0.05.

## Results

### Demographic and Disease Characteristics of Study Participants

We include 256 epilepsy patients with a normal MMSE score and without any other disease, among whom 114 patients were performed HAMD and DTI assessments. All patients were Han Chinese. Demographic and clinical characteristics of all epilepsy patients were shown in Table 1.

**Table 1:** Demographic and disease characteristics of epilepsy patients.

	**All patients**	**Patients with DTI**
Number	256	114
Gender	Male: 130; Female: 126	Male: 57 Female: 57
Age (year)	28.96 ± 10.25(16–64); 26 [21,34]	29.76 ± 9.82(16–60); 28 [22.75,35]
**Socioeconomic status**		
Marriage		
Unmarried	115	55
Married	138	58
Divorced	3	1
Education (year)	12.04 ± 3.66	12.08 ± 3.90
< High school	80	35
High school	88	36
College or higher	88	43
Employment		
Unemployed	78	32
Student	51	15
Employed	127	67
Income		
Below minimum living guarantee	131	49
Above minimum living guarantee	125	65
Age of onset (year)	19.06 ± 10.23; 18 [12,25]	20.07 ± 10.61; 19 [12,25]
Disease duration (year)	9.92 ± 10.03; 7 [2,15]	9.69 ± 9.67; 6 [3,14]
Seizure classification		
Generalized tonic-clonic	135	57
Absence	2	1
Myoclonic	7	2
Focal onset (focal to bilateral tonic-clonic)	112	54
Epilepsy classification		
GGEs	112	46
GTCS seizures on awakening	10	4
JME	7	2
JAE	2	1
Other GGEs	93	39
Others	144	68
Medical treatment		
No AEDs	44	14
Monotherapy	143	72
Polytherapy	69	28
Frequency		
≥1/week	53	19
≥1/month	80	37
≥1/year	83	33
<1/year	40	25
MMSE score	28.86 ± 2.05	28.66 ± 2.11
NHS3 score	12.19 ± 5.53	11.60 ± 5.38

*GGEs, genetic generalized epilepsies; GTCS, generalized tonic-clonic seizures; JME, juvenile myoclonic epilepsy; JAE, juvenile absence epilepsy; AEDs, antiepileptic drugs; MMSE, Mini-mental state examination; NHS3, National Hospital Seizure Severity Scale*.

### Felt Stigma, Quality of Life, Depression, Personality, and Frontotemporal Contact Fibers Assessment in Epilepsy Patients

We evaluated the stigma, personality, depression, and quality of life traits by corresponding scales. Notably, we found a high incidence of felt stigma in epilepsy patients. There were about 39.8% (102 / 256) patient's felt stigma, with Score 1 in 17.2% (44 / 256), Score 2 in 14.5% (37 / 256) and the highest score (Score 3) present in 8.2% (21 / 256) patients. The other 60.2% (154 / 256) answered “NO” to all three questions. The distribution of answers to each question was shown in Table 2.

**Table 2:** The percentage of different answers to each question of stigma scale.

	**No**	**Yes**
I feel some people are uncomfortable with me because of my epilepsy.	82.0% (210/256)	18.0% (46/256)
I feel some people treat me like an inferior person because of my epilepsy.	68.8% (176/256)	21.2% (80/256)
I feel some people would prefer to avoid me because of my epilepsy.	78.5% (201/256)	21.5% (55/256)

Personality traits were evaluated by EPQ scale, and the average scores were 48.55 ± 12.70, 50.87 ± 12.53, 51.35 ± 12.57, 48.00 ± 10.03 for psychoticism, extraversion, neuroticism and lying subscales, respectively. The average scores of HAMD scale in epilepsy patients was 5.04 ± 5.63 [3(1,7)]. We measured quality of life in epilepsy patients using QOL-31. The total QOL-31 score was 60.90 ± 20.00, with seizure worry for 51.72 ± 25.54, overall QOL for 67.92 ± 27.70, emotion wellbeing for 60.93 ± 20.00, energy-fatigue for 57.02 ± 20.20, cognitive function for 58.25 ± 22.74, medical effects for 51.59 ± 28.90 and social function for 66.64 ± 25.22.

Using DTI, we estimated frontotemporal contact fibers by detecting FA values of UF (Right 0.38 ± 0.06; Left 0.39 ± 0.05), AF (Right 0.47 ± 0.06; Left 0.47 ± 0.07), AC 0.38 ± 0.10, Fornix (Right 0.42 ± 0.10; Left 0.41 ± 0.09), SLF (Right 0.49 ± 0.07; Left 0.46 ± 0.07) and CB (Right 0.54 ± 0.07; Left 0.58 ± 0.08).

### Factors Correlated to Felt Stigma in Epilepsy Patients

Patient's felt stigma were found to have shorter education years (*P* < 0.05), longer duration (*P* < 0.05), and younger age of onset (*P* < 0.01). Moreover, stigmatized epilepsy patients manifested higher HAMD score (*P* = 0.001), higher psychoticism (*P* < 0.01) and neuroticism (*P* < 0.001) score of EPQ scale, and lower QOL-31 score (*P* < 0.001). No differences were found between epilepsy patients with and without stigma in age, gender, marriage, income, employment, seizure frequency, numbers of AEDs application, MMSE, and NHS3 scores (*P* > 0.05) (Tables 3, 4, Supplementary Tables 1, 2).

**Table 3:** Relations between felt stigma and clinical characteristics.

	**Not stigmatized (*n* = 154)**	**Stigmatized (*n* = 102)**	***P*-value**
Sex			0.899
Male	60.8%(79/130)	39.2%(51/130)	
Female	59.5%(75/126)	40.5%(51/126)	
Age	29.21 ± 10.73	28.58 ± 9.52	0.631
Epilepsy classification			0.607
GGEs	58.0%(65/112)	42.0%(47/112)	
Others	61.8%(89/144)	38.2%(55/144)	
Seizure classification			0.701
Generalized seizure	59.0%(85/144)	41.0%(59/144)	
Focal seizure	61.6%(69/112)	38.4%(43/112)	
Duration	8.68 ± 9.61	11.80 ± 10.41	0.015^*^
Age of onset	20.56 ± 10.29	16.80 ± 9.76	0.004^**^
Frequency			0.314
≥1/week	50.9%(27/53)	49.1%(26/53)	
≥1/month	60.0%(48/80)	40.0%(32/80)	
≥1/year	61.4%(51/83)	38.6%(32/83)	
<1/year	70.0%(28/40)	30.0%(12/40)	
NHS3 score	11.79 ± 5.65	12.79 ± 5.33	0.154
AEDs treatment			0.209
No AEDs	68.2%(30/44)	31.8%(14/44)	
Monotherapy	61.5%(88/143)	38.5%(55/143)	
Polytherapy	52.2%(36/69)	47.8%(33/69)	
Socioeconomic status			
Marriage			0.306
Married	56.5%(65/115)	43.5%(50/115)	
Single	63.1%(89/141)	36.9%(52/141)	
Education			0.017^*^
< High school	47.5%(38/80)	52.5%(42/80)	
High school	63.6%(56/88)	36.4%(32/88)	
College or higher	68.2%(60/88)	31.8%(28/88)	
Education (year)	12.46 ± 3.81	11.43 ± 3.37	0.028^*^
Employment			0.975
Unemployed	60.3%(47/78)	39.7%(31/78)	
Student	58.8%(30/51)	41.2%(21/51)	
Employed	60.6%(77/127)	39.4%(50/127)	
Income			0.899
Below minimum living guarantee	59.5%(78/131)	40.5%(23/131)	
Above minimum living guarantee	60.8%(76/125)	39.2%(49/125)	
Eysenck personality scale			
Psychoticism score	46.67 ± 14.19	51.32 ± 9.54	0.004^**^
Extraversion score	52.03 ± 12.30	49.16 ± 12.73	0.074
Neuroticism score	46.57 ± 11.83	58.38 ± 10.10	0.000^***^
Lying score	48.30 ± 10.11	47.55 ± 9.94	0.561
QOL-31			
Total QOL-31 score	68.71 ± 12.98	49.33 ± 15.75	0.000^***^
Seizure worry	62.04 ± 20.75	36.45 ± 24.37	0.000^***^
Overall QOL	73.41 ± 30.45	59.80 ± 20.66	0.000^***^
Emotional wellbeing	67.31 ± 17.67	51.49 ± 19.60	0.000^***^
Energy/fatigue	63.48 ± 17.51	47.45 ± 20.21	0.000^***^
Cognitive functioning	65.25 ± 19.59	47.89 ± 23.20	0.000^***^
Medication effects	62.29 ± 24.63	35.76 ± 27.55	0.000^***^
Social functioning	77.48 ± 20.48	50.59 ± 22.98	0.000^***^

*GGEs, genetic generalized epilepsies; AEDs, antiepileptic drugs; NHS3, National Hospital Seizure Severity Scale; QOL, personality and quality of life*.

* *P < 0.05*;

** *P < 0.01*;

*** *P < 0.001*.

**Table 4:** Relations between felt stigma and HAMD, FA values of frontotemporal contact fibers.

	**Not stigmatized (*n* = 66)**	**Stigmatized (*n* = 48)**	***P*-value**
HAMD score	3.44 ± 3.78	7.25 ± 6.89	0.001^***^
UF-Right	0.38 ± 0.06	0.39 ± 0.05	0.847
Left	0.39 ± 0.05	0.39 ± 0.06	0.653
AF-Right	0.48 ± 0.06	0.46 ± 0.06	0.126
Left	0.48 ± 0.06	0.46 ± 0.08	0.092
AC	0.39 ± 0.10	0.36 ± 0.10	0.081
Fornix-Right	0.44 ± 0.10	0.40 ± 0.11	0.046^*^
Left	0.42 ± 0.09	0.39 ± 0.09	0.157
SLF-Right	0.51 ± 0.07	0.48 ± 0.07	0.034^*^
Left	0.46 ± 0.07	0.44 ± 0.07	0.097
CB-Right	0.55 ± 0.06	0.54 ± 0.08	0.356
Left	0.60 ± 0.07	0.56 ± 0.08	0.011^*^

*HAMD, Hamilton Depression Scale; UF, uncinate fasciculus; AF, arcuate fasciculus; AC, anterior commissure; SLF, superior longitudinal fasciculus; CB, cingulum bundle*.

* *P < 0.05*;

*** *P < 0.001*.

We further explored the correlation between stigma score and clinical variables, personality, emotion and quality of life traits by Spearman correlation analysis. We performed analysis between stigma scores and age of the patients, disease duration, age of onset, numbers of AEDs, frequency of epilepsy, MMSE score, NHS3 score, education, HAMD score, EPQ score and QOL-31 score. Stigma scores were significantly negatively correlated with education (*r* = −0.167, *P* = 0.008), age of onset (*r* = −0.134, *P* = 0.032), extraversion score of EPQ (*r* = −0.182, *P* = 0.004), total and all the subscale QOL scores (Total QOL: *r* = −0.606, *P* = 0.000; Seizure worry: *r* = −0.520, *P* = 0.000; Overall QOL: *r* = −0.303, *P* = 0.000; Emotional wellbeing: *r* = −0.470, *P* = 0.000; Energy-fatigue: *r* = −0.390, *P* = 0.000; Cognitive functioning: *r* = −0.389, *P* = 0.000; Medication effects: *r* = −0.479, *P* = 0.000; Social functioning: *r* = −0.596, *P* = 0.000), and positively correlated with duration (*r* = 0.170, *P* = 0.006), HAMD score (*r* = 0.361, *P* = 0.000), neuroticism score of EPQ (*r* = 0.501, *P* = 0.000). Other variables did not display significant correlation.

FA values of frontotemporal contact fibers were summarized in Table 4. We found decreased FA values of the right fornix, right SLF, and left CB in stigmatized epilepsy patients. Spearman correlated analysis showed negative correlations between stigma scores and FA values of right SLF (*r* = −0.187, *P* = 0.046) and left CB (*r* = −0.240, *P* = 0.010).

### Felt Stigma Correlated to Left Cingulum, Social Function, and Neuroticism Independently

We carried out logistic regression to identify the most notable impact factor of stigma. We set stigma score as a dependent variable, and age, gender, marriage, income, employment, education, disease duration, age of onset, numbers of AEDs, seizure frequency, seizure type and MMSE, NHS3, HAMD, EPQ, QOL-31 scores and FA values of frontotemporal fibers as independent variables. The results showed that FA value of left CB [*P* = 0.011; OR = 0.000 (95% CI = 0.000–0.163)], social function [*P* = 0.000; OR = 0.935 (95% CI = 0.906–0.964)] of QOL, and neuroticism score of EPQ [*P* = 0.033; OR = 1.123 (95% CI = 1.010–1.249)] were significant factors included in the regression model of stigma scores (Table 5).

**Table 5:** Factors correlated to felt stigma by logistic regression analysis.

	**OR**	**95% CI**	***P*-value**
FA of left cingulum	0.000	0.000–0.163	0.011^*^
Social function of QOL	0.935	0.906–0.964	0.000^***^
Neuroticism	1.123	1.010–1.249	0.033^*^

*FA, fractional anisotropy; QOL, personality and quality of life*.

* *P < 0.05*;

*** *P < 0.001*.

## Discussion

Stigma is one of the common features of epilepsy in both developed and developing countries. It can cause serious harm to the physical, mental, and social wellbeing of a person with epilepsy.

In this study, we found that about 39.8% of epilepsy patients showed felt stigma. Similarly, previous epidemiological surveys showed the incidence of stigmatized persons was 30~62.5% in European (35, 41). In China, about 40% of people with epilepsy did not tell others about their disease (which could be a symbol of stigma), and 36% thought that people treated them differently because of their epilepsy (8).

Previous studies adopted a variety of scales to evaluate the degree of social stigma in epilepsy patients. In the 1980's, a 21-item stigma scale was used (42), and in these years, scales such as internalized stigma of mental illness (ISMI) scale (43), the original or modified version of the Parent/Child Stigma Scale developed by Austin et al. (44, 45), the impact of epilepsy scale by Jacoby et al. (46), were designed, modified and adapted. In this study, we use a three-item felt stigma scale, which was one of the most widely used stigma scales and adapted in large-scale epidemiological investigations (35). It is a brief and fast questionnaire in that patients were asked three questions for “yes” or “no” answers, and could be applied to a wide population.

Various factors might be contributing to the stigma of epilepsy. Felt stigma was linked to higher seizure frequency, recency of seizures, younger age at epilepsy onset or longer duration, lower educational level, poorer knowledge about epilepsy, and younger age (47). Consistently, we found shorter education years, longer duration, and younger age of onset might have impacts on felt stigma. Previous studies demonstrated that perceived stigma was shown to vary inversely with age, with younger groups tending to feel more stigmatized compared to the older population (47). And younger age was significantly associated with greater perceptions of stigma in patients with 9 and 14 years of age (19). No relationship was found between felt stigma and age, sex, wealth, seizure type/frequency in this study. We failed to detect differences in stigma scores among three groups (16–20y, 21–44y, and 45–65y) either. The variability of age enrollment might be the explanation for the uncorrelated with age in this study. The presence of seizures emerged as the most common factor associated with higher degrees of perceived stigma, with stigma increasing with seizure severity (19, 47, 48). Our results indicated a similar tendency in patients with seizure frequency less than once per year, compared to those with more than once per week. As previously mentioned, felt stigma in epilepsy is associated with depression (24, 49) and negatively impacts on quality of life (23, 24, 50). We reported the degree of depression and all aspects of quality of life, especially social function had significant correlations to felt stigma as well. In a study of public attitudes toward epilepsy in the United Kingdom, more than a fifth of people questioned agreed with a statement that “people with epilepsy have more personality problems than others” (51). Several studies have demonstrated that perceived stigma is a critical factor for interictal aggression and neurotic personality in people with epilepsy (12, 52–54). Other researchers reported that introverted personality was independently associated with the felt stigma of epilepsy (55). In our results, although both the neuroticism and extraversion of personality were associated with stigma scores, we found that neurotic personality was a more notable aspect of felt stigma through logistic regression analysis.

The underlying neurobiology of stigmatization in epilepsy is still unclear. Some preliminary data illuminated the neural underpinnings of stigma. Krendl et al. (56) investigated the functional anatomic using fMRI and reported the subcortical (e.g., amygdala) and cortical responses (e.g., lateral PFC and anterior cingulate) were correlated to stigma perception. Raij et al. (57) showed stigma resistance in psychiatric patients was associated with rostro-ventral medial prefrontal cortex. Nakamura et al. (58) indicated superficial-intracalcarine cortex connectivity was associated with a better response to the anti-stigma interventions. In epilepsy patients, white matter abnormalities have been identified in several DTI studies (59–61). In our previous study, we also found decreased FA value in the bilateral UF, CB, AF (30). The integrity of white matter, especially frontotemporal contact fibers, was associated with psychiatric symptoms, such as depression (62) and personality disorders (30). In this study, we found a possible relationship between felt stigma and FA values of the right fornix, right SLF, and left CB. CB is a bundle of axons projecting from the posterior cingulate gyrus to the entorhinal cortex and the hippocampus is a terminal node of the inferior CB pathway. The impairment of CB was reported to be contributed to neuroticism (63, 64), depression (65), and schizophrenia (66). In this study, we enrolled the patients with epilepsy of genetic or unknown etiology to avoid confounding impacts on DTI and psychosocial factors from the lesions themselves. Our logistic regression results revealed that left CB might be an independent risk predictor of stigmatization in epilepsy patients.

However, the current study has some limitations. Only epilepsy patients with genetic and unknown etiology were included in this study. We only focused on the impairment of frontotemporal contact fibers and its correlation to stigmatization. The changes of other whiter matter required further study. In this study, we only investigate the “felt stigma,” where the other aspect- “enacted stigma” was not studied. In this study, only 114 cases were evaluated HAMD and DTI, which were performed as the subjects in the last regression analysis. This study preliminarily exhibited the neural correlates of felt stigma in epilepsy patients. Further studies are required to understand underlying neurobiology in stigmatization of epilepsy.

## Conclusion

The current study illuminated a high prevalence of felt stigma in epilepsy patients in China. Felt stigma was found to be significantly associated with neurotic personality and social function of the quality of life. Felt stigma was also crucially related to the integrity of the left cingulum. The results pointed out the demanded social supporting caregivers for people with epilepsy and the necessity of screening for psychiatric symptoms in chronic epilepsy courses. The relationship between white matter and felt stigma suggested a new direction for future studies to free epilepsy patients from stigma.

## Data Availability Statement

The raw data supporting the conclusions of this article will be made available by the authors, without undue reservation.

## Ethics Statement

The studies involving human participants were reviewed and approved by Ethics Committee, Zhongshan Hospital. The patients/participants provided their written informed consent to participate in this study.

## Author Contributions

LM: study concept and design, enrolment of the study participants, and study coordination. KW: data analysis, interpretation, and revising the manuscript. QZ: acquisition of MRI data. JW: acquisition of clinical data. YZ: acquisition of data. WP: assessment of clinical scales. JD: study concept and design, interpretation of data, and revising the manuscript for content. All authors contributed to the article and approved the submitted version.

## Conflict of Interest

The authors declare that the research was conducted in the absence of any commercial or financial relationships that could be construed as a potential conflict of interest.

## Publisher's Note

All claims expressed in this article are solely those of the authors and do not necessarily represent those of their affiliated organizations, or those of the publisher, the editors and the reviewers. Any product that may be evaluated in this article, or claim that may be made by its manufacturer, is not guaranteed or endorsed by the publisher.
