# Supplementary material for: Felt Stigma and Its Underlying Contributors in Epilepsy Patients
Source: Front Public Health. 2022 Apr 26;10:879895. doi: 10.3389/fpubh.2022.879895 (PMC9087196; doi:10.3389/fpubh.2022.879895)
Supplement: Supplementary file 1 [file Data_Sheet_1.pdf]

Supplementary Material

1 Supplementary Tables

Table S1 Relations between the scores of stigma subscales and the clinical characteristics

	Stigma Score				*P* value	Stigmatized	*P* value
	0	1	2	3			
**Sex**					0.835		0.899
Male	60.8%（79/130）	18.5%（24/130）	13.8%（18/130）	6.9%（9/130）		39.2%（51/130）	
Female	59.5%（75/126）	15.9%（20/126）	15.1%（19/126）	9.5%（12/126）		40.5%（51/126）	
**Epilepsy Classification**					0.657		0.607
GGEs	58.0%（65/112）	20.5%（23/112）	13.4%（15/112）	8.0%（9/112）		42.0%（47/112）	
Others	61.8%（89/144）	14.6%（21/144）	15.3%（22/144）	8.3%（12/144）		38.2%（55/144）	
**Seizure Classification**					0.682		0.701
Generalized Seizure	59.0%（85/144）	18.1%（26/144）	13.2%（19/144）	9.7%（14/144）		41.0%（59/144）	
Focal Seizure	61.6%（69/112）	16.1%（18/112）	16.1%（18/112）	6.3%（7/112）		38.4%（43/112）	
**Frequency**					0.257		0.314
>=1/week	50.9%（27/53）	15.1%（8/53）	24.5%（13/53）	9.4%（5/53）		49.1%（26/53）	

>=1/month	60.0%（48/80）	18.8%（15/80）	11.3%（9/80）	10.0%（8/80）		40.0%（32/80）	
>=1/year	61.4%（51/83）	21.7%（18/83）	12.0%（10/83）	4.8%（4/83）		38.6%（32/83）	
<1/year	70.0%（28/40）	7.5%（3/40）	12.5%（5/40）	10.0%（4/40）		30.0%（12/40）	
**AEDs Treatment**					0.687		0.209
No AEDs	68.2%（30/44）	15.9%（7/44）	9.1%（4/44）	6.8%（3/44）		31.8%（14/44）	
Monotherapy	61.5%（88/143）	15.4%（22/143）	15.4%（22/143）	7.7%（11/143）		38.5%（55/143）	
Polytherapy	52.2%（36/69）	21.7%（15/69）	15.9%（11/69）	10.1%（7/69）		47.8%（33/69）	
**Socioeconomic Status**							
Marriage					0.526		0.306
Married	56.5%（65/115）	16.5%（19/115）	17.4%（20/115）	7.6%（11/115）		43.5%（50/115）	
Single	63.1%（89/141）	17.7%（25/141）	12.1%（17/141）	7.1%（10/141）		36.9%（52/141）	
Education					0.044*		0.017*
<High school	47.5%（38/80）	18.8%（15/80）	21.3%（17/80）	12.5%（10/80）		52.5% (42/80)	
High school	63.6%（56/88）	19.3%（17/88）	13.6%（12/88）	3.4%（3/88）		36.4% (32/88)	

College or higher	68.2%（60/88）	13.6%（12/88）	9.1%（8/88）	9.1%（8/88）		31.8% (28/88)	
Employment					0.087		0.975
Unemployed	60.3%（47/78）	16.7%（13/78）	10.3%（8/78）	12.8%（10/78）		39.7%（31/78）	
Student	58.8%（30/51）	27.5%（14/51）	9.8%（5/51）	3.9%（2/51）		41.2%（21/51）	
Employed	60.6%（77/127）	13.4%（17/127）	18.9%（24/127）	7.1%（9/127）		39.4%（50/127）	
Income					0.189		0.899
Below minimum living guarantee	59.5%（78/131）	20.6%（27/131）	10.7%（14/131）	9.2%（12/131）		40.5%（23/131）	
Above minimum living guarantee	60.8%（76/125）	13.6%（17/125）	18.4%（23/125）	7.2%（9/125）		39.2%（49/125）	

GGEs, genetic generalized epilepsies.

* $P < 0.05$

Table S2 Felt stigma in different age groups

	Not stigmatized	Stigmatized
**Age group**		
16-20y	59.3%（35/59）	40.7%（24/59）
21-44y	59.4%（98/165）	40.6%（67/165）
45-65y	59.3%（16/27）	40.7%（11/27）
